# Pan-genome analysis and expression verification of the maize ARF gene family

**DOI:** 10.3389/fpls.2024.1506853

**Published:** 2025-02-11

**Authors:** Quan-cai Man, Yan-qun Wang, Shun-juan Gao, Zhi-chang Gao, Zheng-ping Peng, Jiang-hui Cui

**Affiliations:** ^1^ College of Agriculture, Hebei Agricultural University, Baoding, China; ^2^ College of Resource and Environmental Sciences, Hebei Agricultural University, Baoding, China

**Keywords:** maize pan-genome, auxin transcription factors, structure variation, selection pressure, transcriptome data analysis

## Abstract

Auxin transcription factors regulate auxin responses and play crucial roles in plant growth, development, and responses to abiotic stress. Utilizing the maize pan-genome data, this study identified 35 ARF family members in maize, comprising 21 core genes, 10 near-core genes, and 4 non-essential genes; no private genes were detected. The construction of a phylogenetic tree using Arabidopsis thaliana revealed that the G3 subfamily comprises the highest number of core genes, with a total of 10, and exhibits relative stability throughout the evolution of maize. The calculation of the Ka/Ks ratios for ARF family members across 26 genomes indicated that, aside from *ARF8* and *ARF11*, which were subjected to positive selection, the remaining genes underwent purifying selection. Analysis of structural variation revealed that the expression level of the *ARF4* gene significantly differed as a result of this variation. Simultaneously, the structural variation also influenced the conserved domain and cis-acting elements of the gene. Further combining the transcriptome data and RT-qPCR found that, The expression levels of ARF family members in maize were higher at the early stage of embryo and grain development, and the expression levels of each member in embryo and grain were complementary, and the *ARF4* plays an important role in abiotic stress. In summary, this study utilizes the maize pan-genome and bioinformatics methods to investigate the evolutionary relationships and functional roles of ARF family members in maize, thereby providing a novel theoretical framework for further research on the maize ARF family.

## Introduction

Auxin is a crucial phytohormone that plays a significant role in plant growth and development, regulation of plant metabolism, and response to abiotic stress (Casanova-Sáez et al., 2021; Gomes and Scortecci, 2021; Verma et al., 2022). Auxin is distributed through tissue transport mechanisms to ensure the proper functioning of signal transduction pathways, thereby regulating the development of the plant root system (Roychoudhry and Kepinski, 2022). Auxin controls cell growth through the regulation of cell wall remodeling enzyme expression in plants, thereby influencing pectin biosynthesis (Jobert et al., 2023). Changes in auxin content and transport in plants can mitigate plant damage caused by salt stress (Ribba et al., 2020).

The TAA/YUC pathway is the most well-defined auxin synthesis pathway and includes two key components, tryptophan aminotransferase (TAA) and Yucca sativa (YUC) flavin-dependent monooxygenase, these two enzymes catalyze the conversion of tryptophan (Trp) to auxin (IAA), and the pathway plays an important role in regulating plant development (Blakeslee et al., 2019). In addition to this, different auxin concentrations lead to different auxin responses (Tan et al., 2021). In plants, auxin signal transduction is divided into classical signaling pathways and non-classical signaling pathways. The classical pathway primarily refers to the widely recognized TIR1/AFB-Aux/IAA-ARF signal transduction system. The ETT-mediated auxin conduction system, discovered in recent years, is referred to as a non-classical signaling pathway (Leyser, 2018; McLaughlin et al., 2021). Nuclear auxin signal transduction primarily occurs via the classical pathway, namely the TIR1/AFB-Aux/IAA-ARF signal transduction system, which relies on three key components: TIR1/AFB, Aux/IAA, and ARF (Morffy and Strader, 2022). TIR1/AFB primarily functions as an auxin receptor, mediating its function by binding to Aux/IAA to form distinct complexes (Powers and Strader, 2020). After Aux/IAA is polyubiquitinated and degraded by the proteasome, ARF releases its inhibition and activates the expression of auxin response genes. ARF is the key regulator of auxin-responsive gene expression (Rienstra et al., 2023; Liu et al., 2024b). ARF mainly contains three domains with different functions: DNA binding domain (DBD), middle region (MR) and C-terminal domain (PB1) (Cancé et al., 2022). ARF can be categorized into three major groups based on function: transcription activator A, and transcription repressors B and C. Various ARF transcription factor types regulate distinct auxin responses (Freire-Rios et al., 2020). Currently, ARF has been identified in many plants, and their functions have been elucidated. 25 ARF family members were identified in rice. RT-pcr results showed that the expression of rice ARF genes was induced by auxin, and these genes play important roles in metabolic pathways and cellular processes in rice (Wang et al., 2007). 67 ARF family members were identified in wheat, which were unevenly distributed in six subfamilies. GO enrichment analysis showed that ARF family members played important roles in the growth and development, metabolic process, and response to auxin stimulation in wheat (Chaudhary et al., 2023).

The previous study have identified 31 ARF family members in maize genome and divided them into six subfamilies. ZmARFs proteins range in length from 462 to 1192 amino acids, the expression of ZmARF gene is regulated by auxin and small RNA. It may play a key role in seed development and germination (Xing et al., 2011). However, traditional gene family identification methods typically rely on a single reference genome, which exhibit notable limitations and are unable to discern variations among distinct reference genomes. With the publication of the maize pan-genome, the presence-absence and structural variation (SV) information between the genomes of different maize strains have been made available, offering enhanced support for gene family analysis and research (Hufford et al., 2021).

Based on 26 maize pan-genomes, this study screened and identified 35 ARF gene family members, including 21 core genes, 10 near-core genes, 4 non-essential genes, and found no private genes. By analyzing the nonsynonymous to synonymous substitution ratios (Ka/Ks) of ARF members in 26 genomes, the effects of structural variation on gene expression, gene structure, and conserved domains were revealed. Further analysis of the transcriptome expression data of ARF family members revealed that ARF is crucial for the development of maize embryos and seeds.

## Materials and methods

### Plant material and stress treatment

Maize inbred line B73 was selected as the experimental material. Maize seeds were sown in black pot (7cm×8cm×5cm) soil. Only one corn was sotted in each pot and then placed in a 25 °C thermostatic chamber for cultivation. When maize grew to the trifoliate stage, maize plants were subjected to simulated drought stress and salt stress (Drought stress:20% PEG6000; Salt stress: 200mM NaCl). Samples were then taken at 12 h and 24 h of drought and salt stress treatments, respectively. The samples were flash frozen in liquid nitrogen and then stored in an ultra-low temperature refrigerator at -80 °C.

### Identification of maize ARF gene family

26 maize pan-genome data sets were derived from the research conducted by Hufford et al (Hufford et al., 2021).Subsequently, the hidden Markov model of ARF (PF06507) was retrieved from the Pfam database (https://www.ebi.ac.uk/interpro/entry/pfam/), and then the ARF domain was searched using HMMER 3.1 software, with the threshold set to 1e ^-5^. The preliminary ARF members were submitted to the SMART (http://smart.embl.de/) and Pfam databases (https://www.ebi.ac.uk/interpro/) for identification, and genes lacking the ARF domain were removed. Ultimately, the maize ARF members were characterized, and the protein lengths of the identified ARF members were evaluated using BioPerl (Stajich et al., 2002).

### ARF presence-absence variation analysis

The presence-absence information of ARF members was derived from the research conducted by Hufford et al (Hufford et al., 2021). Subsequently, a built-in script was utilized to generate the list of presence-absence genes, and the ggplot2 package in R scripts was employed to create the presence-absence heat map of ARF members in 26 genomes (Ito and Murphy, 2013).

### Phylogenetic analysis

Arabidopsis protein sequences were retrieved from the Arabidopsis database, and the conserved domain information for Arabidopsis ARF family members was extracted. Then, muscle was used to align the conserved domains of maize and Arabidopsis ARFs, and then the maximum likelihood method (Maximum Likelihood, Bootstrap repeated 1000 times) was used to construct the phylogenetic tree of Arabidopsis and maize ARF family members through IQTREE (Nguyen et al., 2015). The resultant evolutionary tree was submitted to ITOL v6 (https://itol.embl.de/) for enhancement and visualization.

### Ka/Ks calculation

The coding sequences (CDS) and protein sequences of ARF family members were retrieved from 26 maize genomes, and the Ka/Ks values for each ARF family member were computed using KaKs Calculator 2.0 (Wang et al., 2010a). Subsequently, the R software packages ggridges and ggplot2 were employed to create the Ridgeline plot of Ka/Ks, and the R software package pheatmap was used to create the heat map for ARF members with Ka/Ks values exceeding 1.

### Analysis of the expression of *ARFs* overlapped with SVs

The structural variation analysis results of 26 maize pan-genomes were derived from the research conducted by Hufford et al (Hufford et al., 2021). the B73 genome was chosen as the reference genome for library construction; subsequently, ANNOVAR was employed to annotate the structural variation (Wang et al., 2010b). Then the information that needs to be retained and structural variation is extracted, and then the structural variation information of ARF family members is extracted. Subsequently, a correlation analysis was performed on the structural variation of ARF family members and gene expression levels, and a histogram was generated for genes exhibiting significant differences.

### Analysis of the SV and gene structure

Download the genome annotation file from the maize database website (http://maize-pange.nome.gramene.org.), then extract the significantly different gene structure information identified in the previous step, and submit the protein sequence to the MEME program (http://meme-suite.org/) for protein conserved motif analysis (parameter settings: the maximum number of motifs is 10, the width of the motif is set to 6-50, and the rest are default parameter values). Use TBtools II to visualize gene structure and conserved motifs (Chen et al., 2023).

Subsequently, the protein sequence of the reference genome B73, along with the protein sequence of the strain exhibiting the greatest overlap with its structural variation (SV), was submitted to the MEME program for conserved motif analysis (parameter settings are the same as above), and webLogos representations of the ARF member protein sequences for both strains were generated.

### Analysis of cis-acting elements of the promoter

The reference genome B73 and the 2000bp promoter sequence of the strain with the most overlap with SV were extracted. The promoter sequences were submitted to the PlantCARE database (http://bioinformatics.psb.ugent.be/webtools/plantcare/html/) for cis-acting element analysis (Lescot et al., 2002). Then, Tbtools II software was used to visualize the cis-acting elements (Chen et al., 2023).

### Protein-protein interaction network analysis of auxin signaling pathway

From phytozome (JGI) database (https://phytozome.jgi.doe.gov) to download the members of the family of IAA corn protein sequences, it was uploaded to the STRING database together with the ARF family member sequences of B73 (Szklarczyk et al., 2023), the interaction between ARF family and IAA family was analyzed, and the results were visualized by cytoscape software (Shannon et al., 2003).

### RNA-seq data analysis

Data were downloaded from the NCBI database based on the RNA-seq data (PRJNA237837), with B73 selected as the reference genome, and Transcripts Per Million (TPM) were utilized as the metric to quantify the expression level. The expression profiles of ZmARF family members in embryos and seeds across various developmental stages were analyzed, and TBtools II was utilized to generate the expression heatmap for ZmARFs (Chen et al., 2023).

### RT-qPCR analysis

Total RNA was extracted from the samples using Promega (LS1040), cDNA was synthesized by reverse transcription using Kangwei Century RT gDNA (CW2020M), RT-qPCR was performed on CFX96 (Bio-Rad, USA) using the US EVERBRIGHT (AugeGreen qPCR Master Mix S2008L) (Three biological replicates and three technical replicates were used). Relative expression was calculated using the 2^-ΔΔct^ method (Maren et al., 2023). The primers used were synthesized by Sangon Bioengineering (Shanghai) Co., LTD.

## Result

### Identification and phylogenetic analysis of ZmARFs gene-based pan-genome

**Figure 1:**
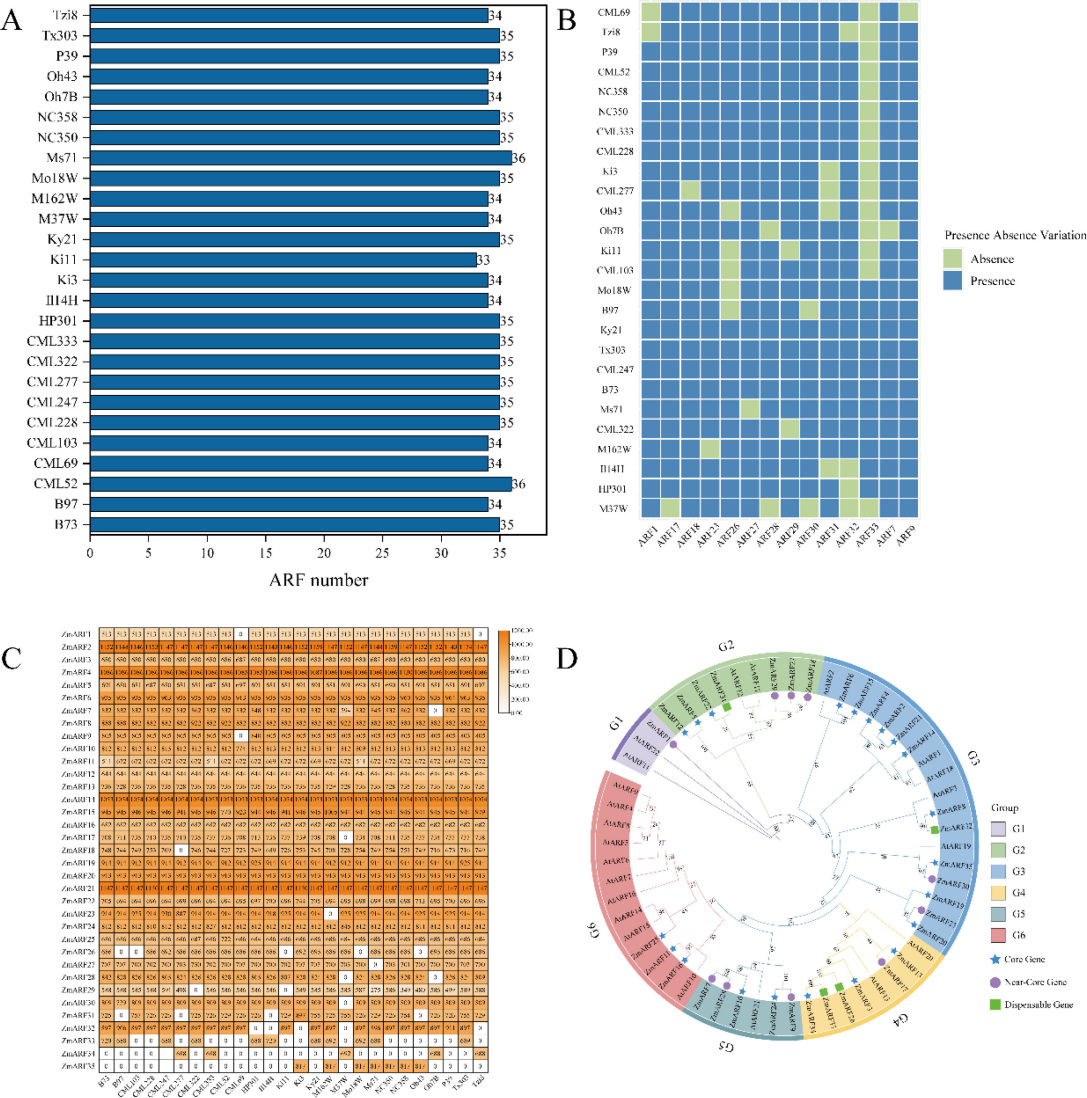
Identification and phylogenetic analysis of ZmARFs in pan-genome. **(A)** Number of ZmARFs. **(B)** Heatmap of the presence and absence of 14 ZmARFs in 26 maize varieties except for the core genes. **(C)** Heatmap of ZmARFs protein length. **(D)** Phylogenetic tree of ARFs from Arabidopsis and Maize.

In total, 35 ZmARF family members were identified from the maize pan-genome. B73 was utilized as the reference genome to compare the quantity of ARF family members and the lengths of protein sequences across various maize lines, revealing significant differences among the lines. Regarding the number of ARF family members, CML52 and MS71 both identified 36 ARF members, the highest number among the lines, whereas Ki1 identified 33 ARF members, the lowest count (**Figure 1A**). The presence-absence analysis of the identified ZmARF family members revealed that the ZmARF family comprises 21 core genes, 10 near-core genes, and 4 non-essential genes. No private genes were identified within the ZmARF family (**Figure 1B**). A further analysis of the protein lengths of ARF family members across different strains indicated that only *ARF12*, *ARF16*, and *ARF20* were consistently present in all strains with identical protein lengths, whereas *ARF1* was absent in CML69 and Tzi8, although the protein lengths of *ARF1* in the other strains remained consistent. With the exception of these four genes, the protein lengths of the other family members exhibited variability (**Figure 1C**). Complete information of ARF family members is provided in **Supplementary Table S1**.

A phylogenetic tree was constructed based on the domain sequences of maize and Arabidopsis and classified into six subtribes according to the study by Xing et al (Xing et al., 2011). The G1 subtribe includes only one near-core gene, the lowest count, while the G3 subtribe comprises 13 ARF family members, the highest count, comprising 10 core genes, 2 near-core genes, and 1 non-essential gene. The G2, G4, G5, and G6 subtribes comprise 7, 6, 5, and 3 ARF family members, respectively, and all ARF family members in the G6 subtribe are core genes (**Figure 1D**).

### ZmARF is subjected to different selection pressures among maize varieties

**Figure 2:**
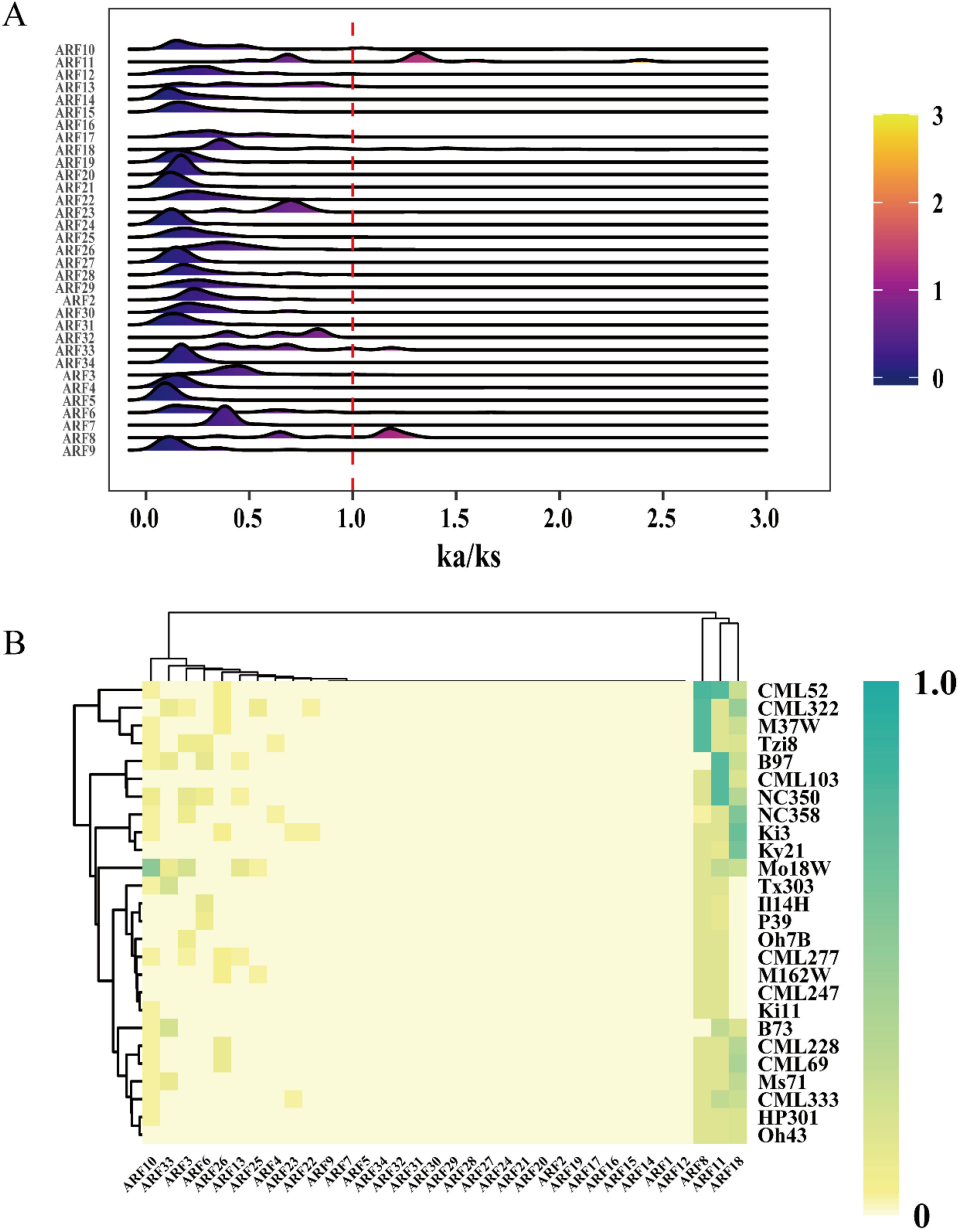
Ka/Ks values of ZmARF. **(A)** A Distribution of Ka/Ks values of ZmARF in 26 maize varieties. **(B)** Heatmap of the frequency of occurrence of different maize varieties at each ARF with Ka/Ks ratio > 1.

The Ka/Ks ratio serves as a significant metric for investigating the mechanisms of genetic evolution (Hurst, 2002). To investigate the selection pressure on ARF family members across 26 maize genomes, the Ka/Ks values for each ARF family member were computed. The results indicated that the Ka/Ks values for *ARF1* and *ARF35* could not be determined. With the exception of the peak values for *ARF8* and *ARF11*, which exceeded 1, the Ka/Ks peak values of the majority of other ARF family members were below 1 (**Figure 2A**). Some maize strains exhibit positive selection effects on *ARF8* and *ARF11*, whereas the other genes are subject to purifying selection. Further analysis of the heatmap for Ka/Ks ratios greater than 1 revealed that only *ARF8*, *ARF11*, and *ARF18* exhibited high Ka/Ks ratios, indicating that these genes were subjected to selection pressure during maize development (**Figure 2B**).

### Expression, structure and motif of ZmARF genes are affected by SV

**Figure 3:**
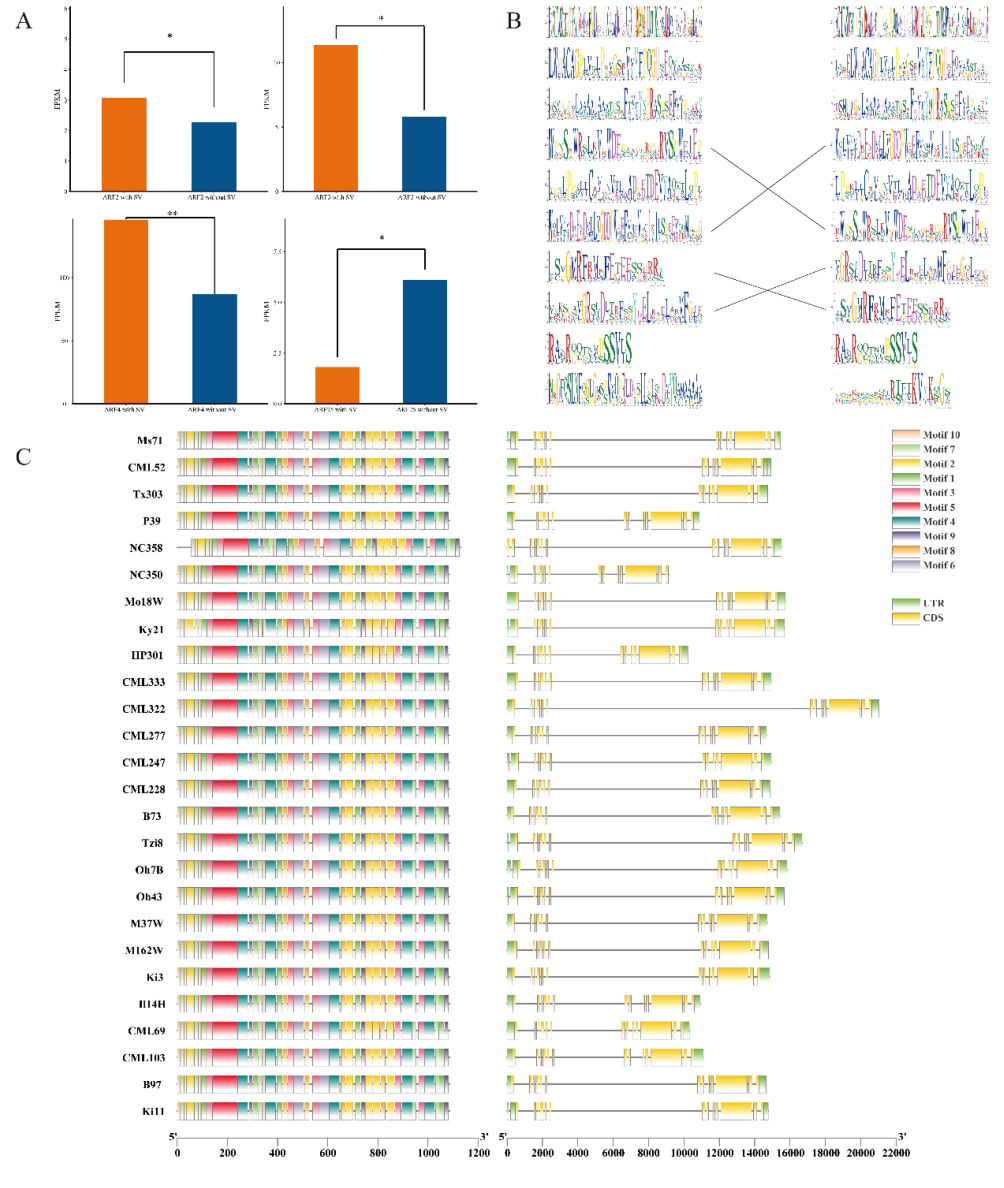
SVs affecting the expression, structure and motif in 26 maize genomes. **(A)** The expression of ARF2,ARF3,ARF4,ARF25 was significantly affected by SVs (*:P<0.05, **:P<0.01). **(B)** A The weblogos of the Ki3 ZmARF and the reference genome are shown on the left and right, respectively. The weblogos connected by the lines indicate that they are corresponding. Weblogos are arranged in the order of E-value. **(C)** The structure and motifs of ZmARF4 in maize pan-genome.

The variation data of ARF family members were extracted from the maize structural variation file; subsequently, the Pearson correlation coefficients of the expression levels of genes both overlapping and not overlapping with SV were calculated. The results indicated that *ARF2*, *ARF3*, *ARF4*, and *ARF25* exhibited significant differences in expression levels between the presence of structural variation (SV) and its absence. These significant differences suggest that SV markedly influences the expression of these four genes (**Figure 3A**). Subsequently, we calculated the number of genes exhibiting structural variation (SV) overlap between the reference genome B73 and other genomes, and selected the strain Ki3, which exhibited the highest degree of overlap, to generate a conserved domain alignment diagram (**Figure 3B**). The results indicated that in the Ki3 strain genome, three conserved domains were identified as corresponding to the reference genome B73, whereas the remaining seven did not align. Furthermore, the amino acids within the corresponding conserved domains of each group do not exhibit complete alignment, suggesting that structural variation (SV) substantially influences the conserved domains of ZmARF. In the gene expression analysis, *ARF4* exhibited the most significant difference; therefore, we delineated its gene structure and conserved domains across 26 maize genomes. Our analysis revealed that the gene structure and conserved domains in the remaining genomes were in alignment with those of the reference genome B73, showing no significant discrepancies (**Figure 3C**).

### Analysis of cis-acting elements of the promoter

**Figure 4:**
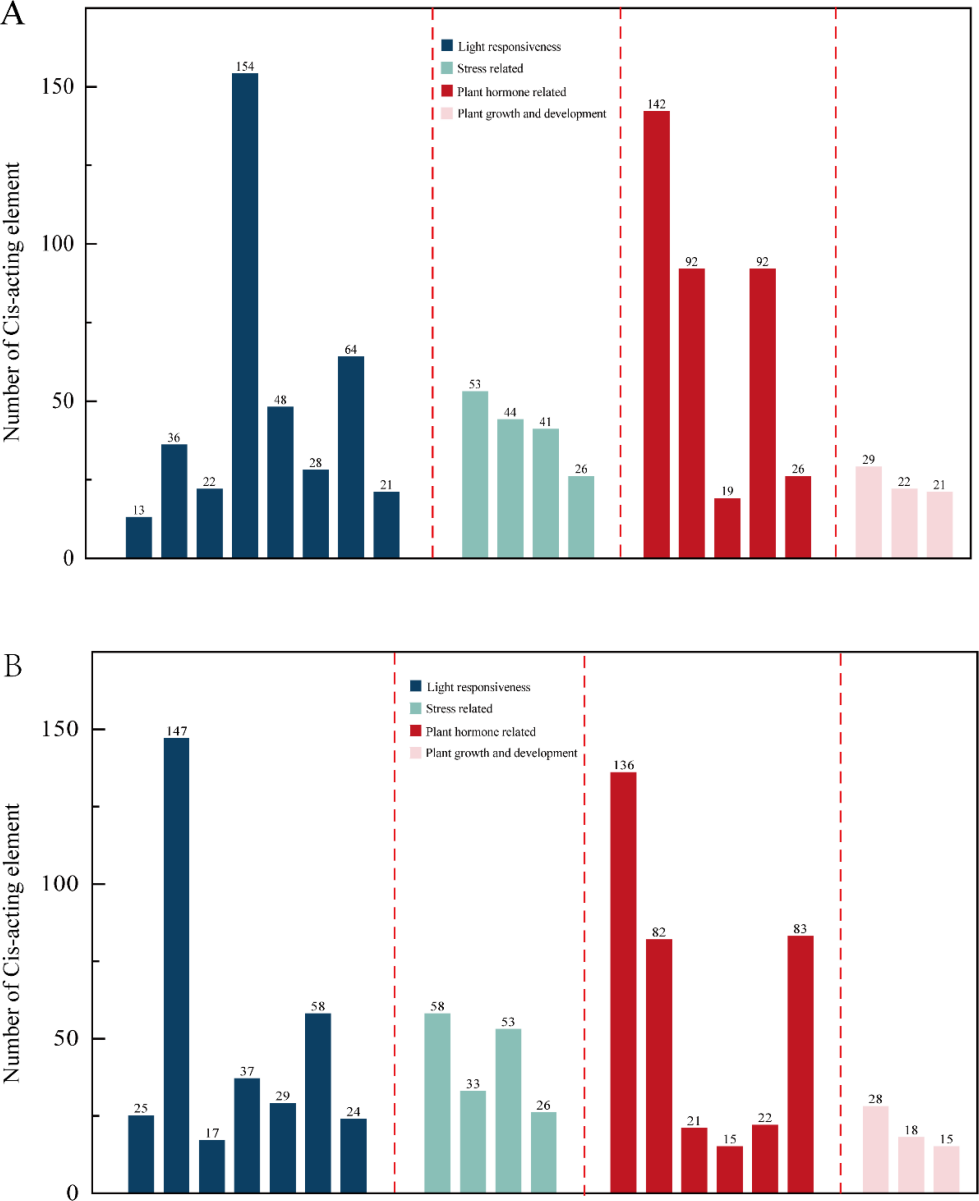
Statistics of the number of cis-acting elements in B73 and Ki3. **(A)** B73 Cis-acting element number statistics. **(B)** Ki3 Cis-acting element number statistics.

The Ki3 strain with the most overlap with the reference genome SV was selected, analysis of cis-acting elements in the promoter, the top 20 elements with the largest number of cis-acting elements were selected for statistics and drawing. It was found that in the reference genome B73 and Ki3, there are differences between cis-acting elements related to light response and cis-acting elements related to plant hormones, B73 contains 8 cis-acting elements related to light response and 5 cis-acting elements related to plant hormones (**Figure 4A**); Ki3 contains 7 cis-acting elements related to light response and 6 cis-acting elements related to plant hormones (**Figure 4B**). Both B73 and Ki3 contained four stress-related cis-acting elements and three growth-related cis-acting elements. It is concluded that structural variation alters the cis-acting element composition of ARF members in different strains, which may further affect physiological processes such as photosynthesis and hormone response in plants. The composition of ARF cis-acting elements in B73 and Ki3 is detailed in **Supplementary Figure S1**.

### IAA-ARF protein-protein interaction network analysis

**Figure 5:**
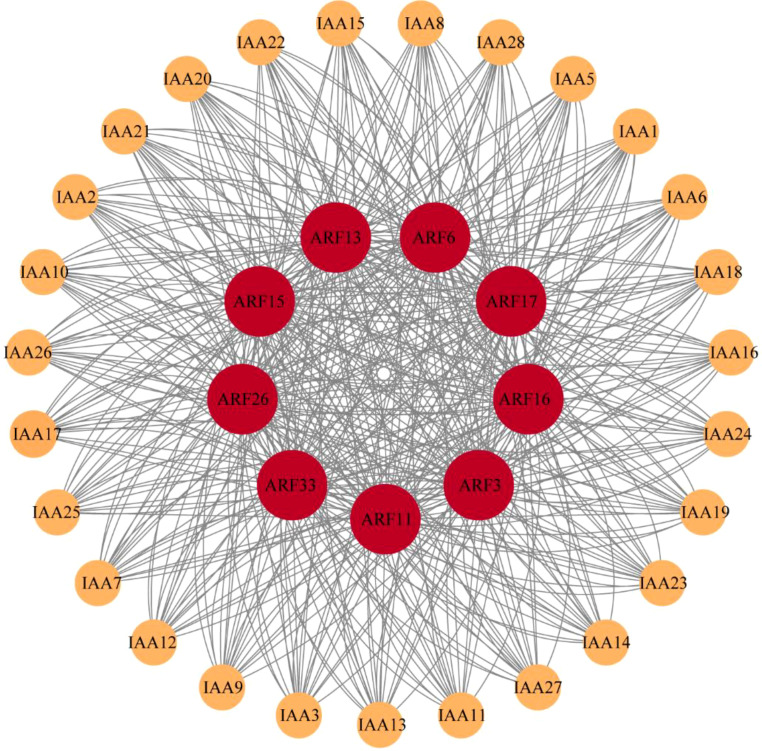
ARF- IAA family protein-protein interaction network.

By analyzing the interaction between IAA family and ARF family in maize, it was found that they were closely related (**Figure 5**). There are 9 members of the ARF family, *ARF3*, *ARF6*, *ARF11*, *ARF13*, *ARF15*, *ARF16*, *ARF17*, *ARF26* and *ARF33*, play key roles in protein-protein interactions with 28 members of the IAA family. It is concluded that these nine members of the ARF family are more closely related to auxin during auxin signaling and thus play an important role in auxin signaling and regulating plant growth and development.

### Effects of ARF on maize seed development

**Figure 6:**
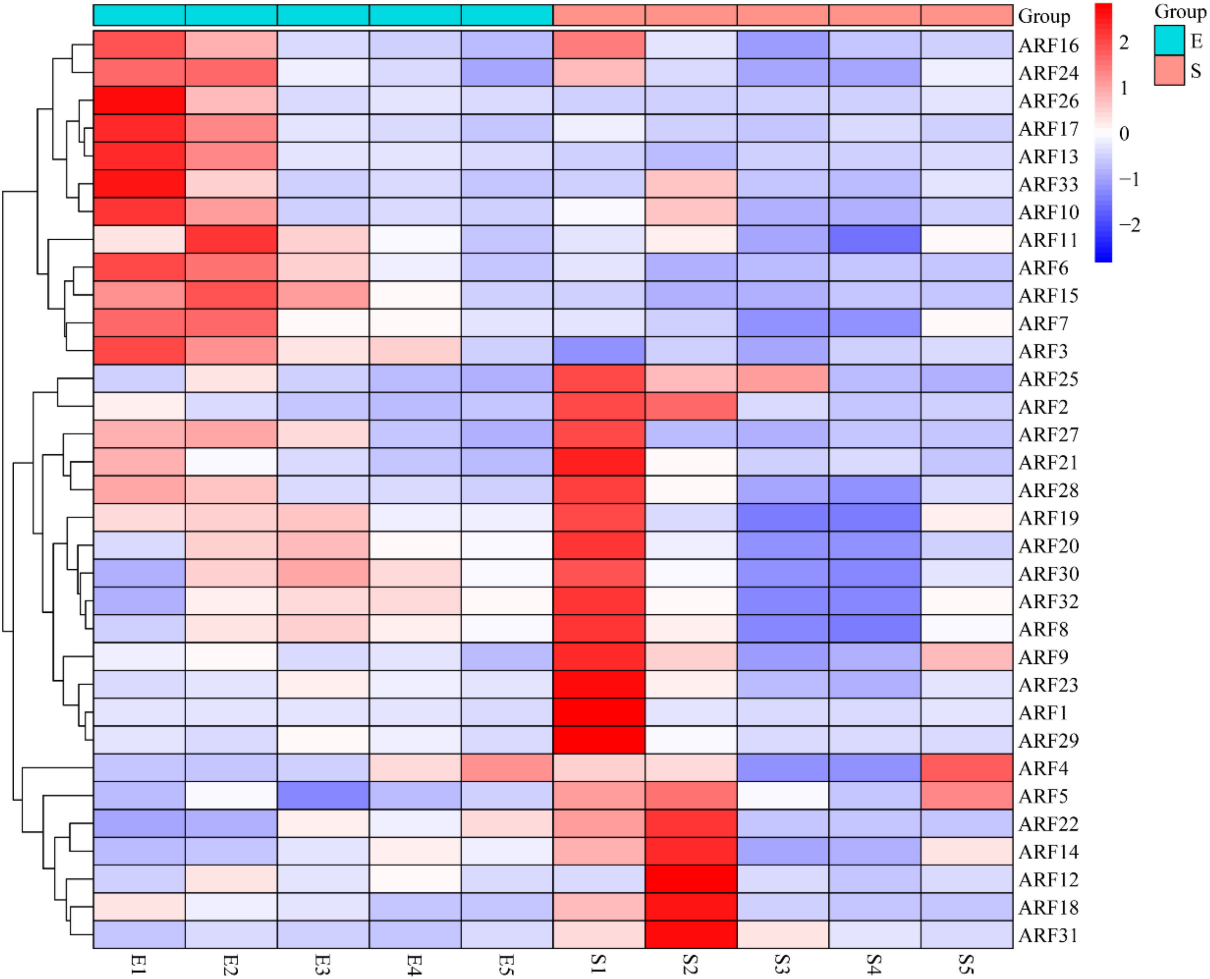
Heatmap of maize embryo and seed development with ARF family members (E1: embryo-10day; E2: embryo-16day; E3: embryo-24day; E4: embryo-30day; E5: embryo-36day; S1: seed-0day; S2: seed-6day; S3: seed-12day; S4: seed-22day; S5: seed-32day).

Transcriptome data pertaining to maize embryos and seeds across various developmental stages were retrieved from the SRA database, and the expression profiles of ARF family members at these stages were subsequently analyzed. The results showed that 11 ARF family members were highly expressed in E1 (embryo-10day), 12 ARF family members were highly expressed in E2 (embryo-16day), 16 ARF family members were highly expressed in S1 (seed-0day), 7 ARF family members were highly expressed in S2 (seed-6day), and only 2 ARF family members were highly expressed in S5 (seed-32day), while the expression of ARF family members in the rest of the developmental stages was at a low level (**Figure 6**). These findings suggest that ARF family members play a crucial role in the early development of maize embryos and seeds.

### ARF in response to abiotic stress

**Figure 7:**
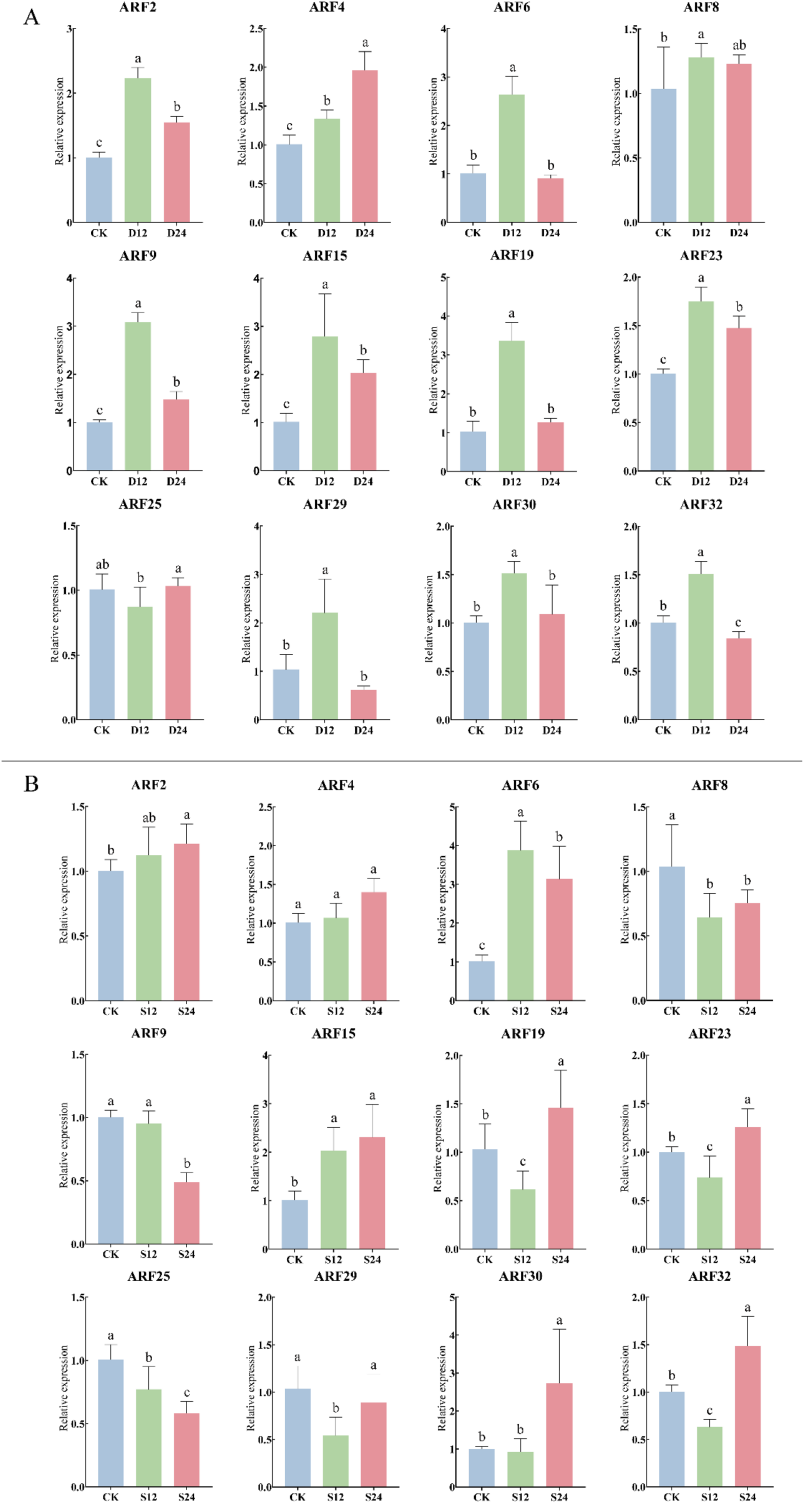
Expression of 12 ARF genes in response to abiotic stress (The horizontal axis represents the different stress treatment times; The vertical axis represents the relative expression of gene). **(A)** Expression of ARF genes under drought stress (P<0.05, The error bars indicate standard deviation. D12, Drought stress for 12 h; D24, Drought stress for 24 h). **(B)** Expression of ARF genes under salt stress (P<0.05, The error bars indicate standard deviation. S12, Salt stress for 12 h; S24, Salt stress for 24 h).

The response of the ZmARF gene to drought and salt stress was further analyzed. The results showed that under 20% PEG6000 drought stress treatment, ZmARF gene was significantly differentially expressed at different treatment times (**Figure 7A**). The expression levels of *ARF2*, *ARF6*, *ARF8*, *ARF9*, *ARF15*, *ARF19*, *ARF23*, *ARF29*, *ARF30* and *ARF32* reached the peak at 12 h of stress, and then decreased to different degrees at 24 h of stress. *ARF4* and *ARF25* peaked at 24 h after stress treatment. Compared with CK, *ARF4* was up-regulated at 12 h of stress, while *ARF25* was down-regulated at 12 h of stress and then up-regulated at 24 h of stress. Under the treatment of 200 mM NaCl, the expression of ZmARF gene was also significantly different under different treatment time (**Figure 7B**). *ARF2* and *ARF6* reached the peak at 12 h, and then were down-regulated at 24 h compared with 12 h. *ARF4*, *ARF15*, *ARF19*, *ARF23*, *ARF30* and *ARF32* peaked at 24 h of stress treatment, Among them, *ARF4* and *ARF15* were up-regulated, while *ARF19*, *ARF23*, *ARF30* and *ARF32* were down-regulated after 12 h of stress compared with CK; The expression levels of *ARF8*, *ARF9*, *ARF25* and *ARF29* were down-regulated under salt stress. Among them, *ARF8* and *ARF29* were up-regulated at 24 h compared with 12 h, while *ARF9* and *ARF25* were gradually down-regulated over time under salt stress. In conclusion, ZmARF members responded to drought stress and salt stress to different degrees. Among them, only *ARF4* expression increased gradually with the increase of stress time under drought stress and salt stress, suggesting that it plays an important role in the response to abiotic stress.

## Discussion

Traditional gene family analysis is usually based on a single reference genome. Given the limitations inherent to a single reference genome, it becomes challenging to ascertain the presence or absence of genes across multiple reference genomes. Following the publication of the maize pan-genome, a more thorough and extensive analysis of the maize gene family can be conducted. The maize pan-genome encompasses the genomes of 26 distinct maize lines constructed using B73 as the reference genome, thereby incorporating genes that are absent from the reference genome. Utilizing the maize pan-genome, this investigation identified 35 members of the maize ARF family. In comparison to the 31 members identified by Xing et al. (Xing et al., 2011) the information regarding ARF family members derived from the pan-genome is more comprehensive. Of the 35 ZmARF family members, only 33 genes were discerned within the reference genome, a phenomenon commonly observed across various species. For instance, the maize pan-genome identified 32 ZmTPS genes, representing an increase of three compared to the 29 genes found in the reference genome (Sun et al., 2023). The number of TPS members present in the rice reference genome is fewer in comparison to the pan-genome (Sun et al., 2022). The PAV analysis revealed that only 21 out of the 35 gene family members were consistently present across all maize varieties. These core genes are likely to play crucial roles in the growth and development of maize. Furthermore, the remaining genes were not universally absent across all varieties, thereby ensuring the complementarity of the genomes among the different varieties.

Gene structural variations typically encompass deletions, insertions, copy number variations (CNVs), inversions, and translocations, which significantly influence plant phenotypic traits, gene expression, and protein functionality (Gabur et al., 2019; Yuan et al., 2021). Furthermore, structural variation significantly influences crop resistance to both biotic and abiotic stresses (Tao et al., 2019). For instance, the structural variation in the rice restorer gene Rf20 enables the restoration of fertility in wild abortive cytoplasmic male sterility (CMS-WA) rice lines (Song et al., 2024); The maize *ZmWUS1* gene promotes the occurrence of duplication events in the *Bif3* mutant. The expression of the *ZmWUS1* gene is inhibited, resulting in altered maize meristem size (Chen et al., 2021). Based on the maize pan-genome structural variation file, this study identified the structural variation information of ARF family members in different genomes. The results indicated significant differences in the expression levels of four genes, namely *ARF2*, *ARF3*, *ARF4*, and *ARF25*. Further verification revealed that structural variation (SV) significantly affects the conserved domain of the gene. Further analysis of structural variants on cis-acting elements revealed that, Structural variants lead to differences in the composition of cis-acting elements between the Ki3 strain and the reference genome B73, may further lead to changes in some physiological activities of ARF family members in these two strains.

Maize kernels are composed of two main parts: the embryo and the endosperm. These kernels typically undergo three maturation processes: early development, filling, and dehydration (Doll et al., 2017). Research has indicated that the development of maize kernels is typically influenced by plant hormones, sugars, receptors, and transcription factors (Li et al., 2021). Auxin, as a vital plant hormone, plays a crucial role in the development of maize kernels. Auxin regulates physiological processes, including plant growth and development, by interacting with auxin response factors (Chen et al., 2020; Cancé et al., 2022). Analysis of transcriptome expression in seeds and embryos at various developmental stages revealed that ZmARF members were highly expressed during the early developmental phase of maize seeds and embryos. ARF family members may function as the initial signal for maize kernel maturation and facilitate the signaling process involved in this maturation. Furthermore, our analysis revealed that during the development of maize embryos and kernels, the highly expressed ARF family members exhibited a complementary relationship; specifically, members that were highly expressed in embryos demonstrated lower expression levels in seeds, while those highly expressed in seeds exhibited lower levels in embryos. Moreover, among the 12 ARF members that were highly expressed in embryos, 8 were identified as core genes; similarly, among the 16 members highly expressed in kernels, 13 were classified as core genes. These findings suggest that ZmARF plays a crucial role in the development of both maize embryos and kernels.

Previous studies have found that ARF family members are involved in plant response to abiotic stress. For example, overexpression of *ZmARF1* can significantly enhance the tolerance of transgenic Arabidopsis plants to abiotic stresses such as low phosphorus stress, drought stress and salt stress (Liu et al., 2024a); Transcriptome studies showed that *ARF10* and *ARF14* were involved in the response of maize to drought stress and were significantly up-regulated under drought stress (Zou et al., 2024). Based on this, further study on the responses of ARF family members to abiotic stress revealed that ARF family members had different responses to drought stress and salt stress. 12 ARF family members were up-regulated under drought stress, 8 members were up-regulated under salt stress, and 4 members were down-regulated under salt stress. In conclusion, ARF members are involved in maize responses to drought stress and salt stress, and ARF members may be more sensitive to drought stress.

In summary, this study provides an in-depth exploration of maize ARF family based on high-quality pan-genome data. The impact of structural variation on the number of ARF family members, gene structure, conserved domains and cis-acting elements in maize was revealed. In addition, the important roles of ARF family members in maize physiological processes such as grain maturation and response to abiotic stress were further elucidated. These results provide effective theoretical support for the growth and development mechanism of maize.

## Data Availability

Publicly available datasets were analyzed in this study. This data can be found here: PRJNA237837.

## Supplementary material

Supplementary Figure 1 Analysis of cis-acting elements of the promoter. **(A)** Cis-acting elements of the B73 promoter. **(B)** Cis-acting elements of the Ki3 promoter.
