# Supplementary figures and images for: Pan-genome analysis and expression verification of the maize ARF gene family
Source: Front Plant Sci. 2025 Feb 11;15:1506853. doi: 10.3389/fpls.2024.1506853 (PMC11850412; doi:10.3389/fpls.2024.1506853)

B73

A

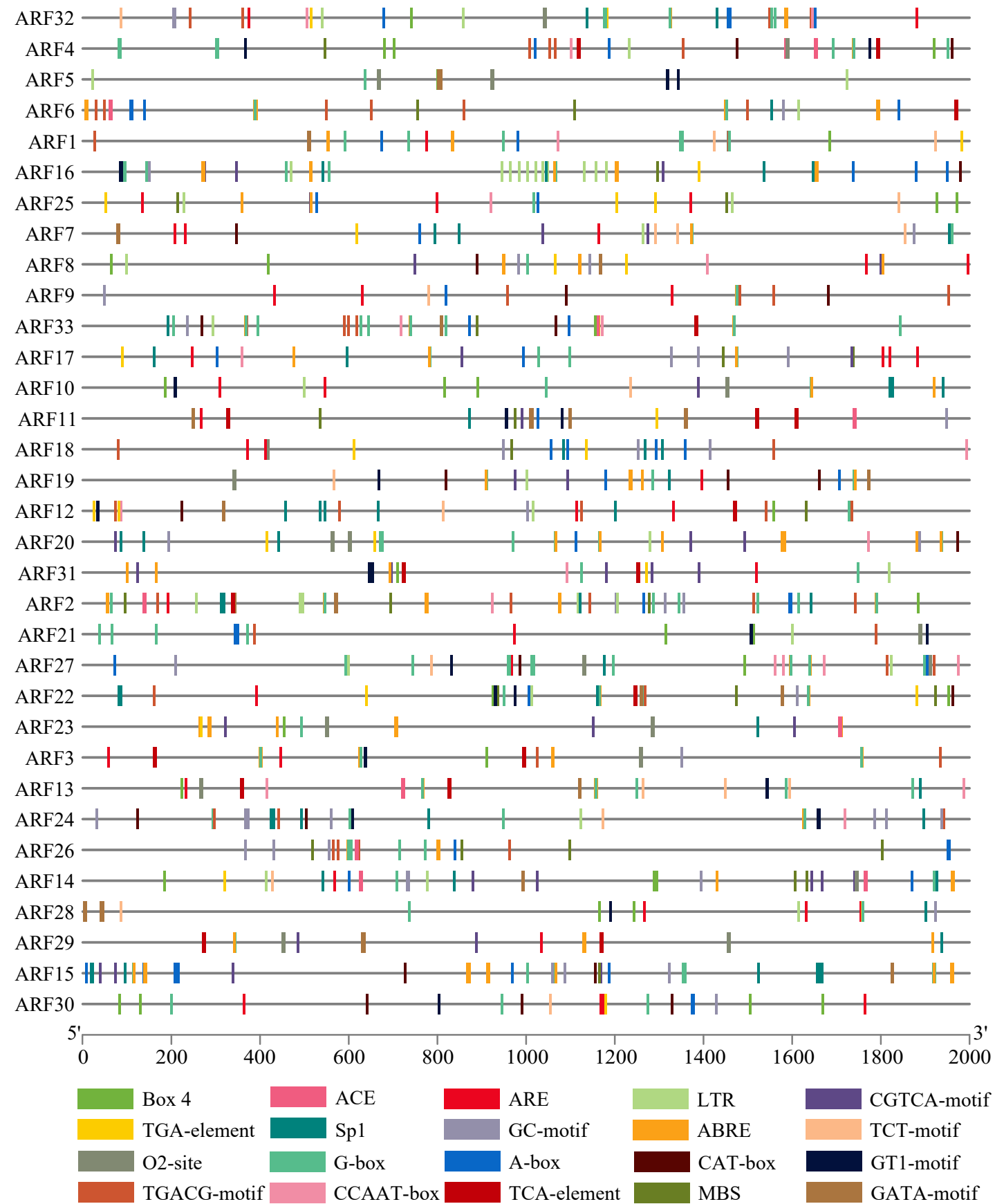

Ki3

B

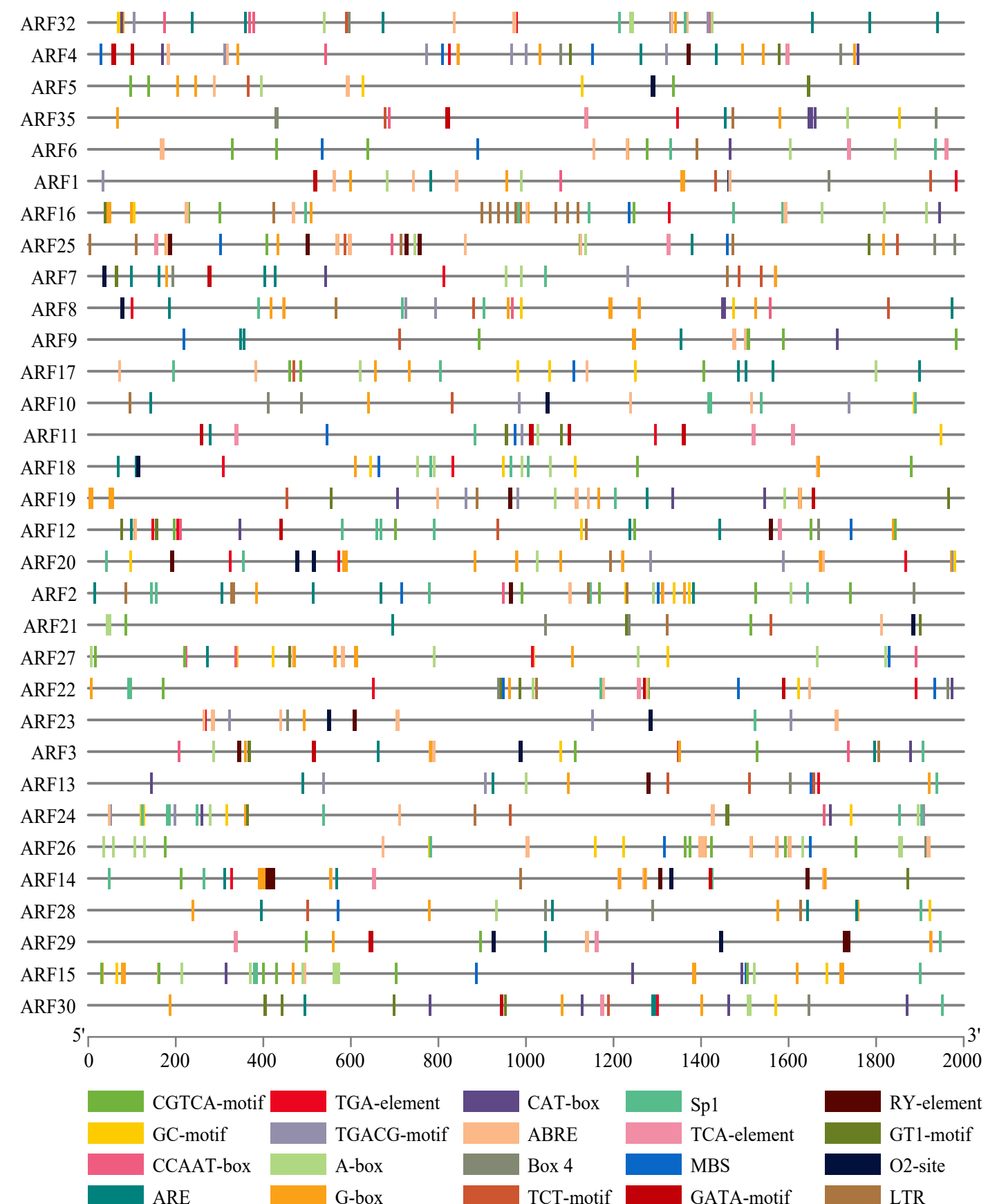

Supplement: Supplementary Figure 1 — Analysis of cis-acting elements of the promoter. (A) Cis-acting elements of the B73 promoter. (B) Cis-acting elements of the Ki3 promoter. [file SupplementaryFile1.pdf]
